# Medication review interventions for adults living with advanced chronic kidney disease: A scoping review

**DOI:** 10.1111/bcp.16363

**Published:** 2024-12-30

**Authors:** Cathy Margaret Pogson, Rosalynn Austin, Jignesh Prakash Patel, David Collins Wheeler

**Affiliations:** ^1^ Pharmacy Department Portsmouth Hospitals University NHS Trust Portsmouth UK; ^2^ Department of Public Health, Faculty of Health Sciences University of Stavanger Stavanger Norway; ^3^ Cardiology Department Portsmouth Hospitals University NHS Trust Portsmouth UK; ^4^ NIHR Applied Research Collaborative Wessex Innovation Centre Southampton UK; ^5^ Institute of Pharmaceutical Science, Faculty of Life Sciences & Medicine Kings College London London UK; ^6^ Department of Haematological Medicine King's College Hospital Foundation NHS Trust London United Kingdom; ^7^ Department of Renal Medicine University College London London UK

**Keywords:** adverse drug reactions, co‐morbidity, deprescribing, medicines optimisation, morbidity

## Abstract

Structured medication reviews (SMRs) were introduced into the National Health Service (NHS) Primary Care to support the delivery of the NHS Long‐Term Plan for medicines optimization. SMRs improve the quality of care, reduce harm and offer value for money. However, evidence to support SMRs for patients with chronic kidney disease (CKD) stage G4‐5D with elevated risk of cardiovascular disease and premature mortality is unknown. This scoping review aimed to assess the extent and nature of SMR research in the population of patients with CKD stage G4‐5D. Electronic databases were searched on 20 October 2023. Studies were eligible if they described an SMR in adults with CKD stage G4‐5D, regardless of the study design. Data detailing the global patterns, population and intervention descriptions, professionals performing SMR, and reported areas for future research were extracted. The extracted outcome data were categorized as clinical, patient‐important, medication‐related and experience‐related. A narrative synthesis was completed. Seventeen studies (81%) were conducted in nephrology outpatient settings, three (14%) during acute hospital admissions and one (5%) within the community pharmacy. Eighteen studies (86%) were quantitative, including five randomized controlled trials. Ten (48%) studies were undertaken in the United States and Canada, and two in Europe (France and Norway). No such studies have been conducted in the United Kingdom. Our review revealed that there is a lack of evidence for SMR as a strategy to reduce polypharmacy and harms from medication for adults with CKD stage G4‐5D. Therefore, further research is required in this area.

## Graphical abstract

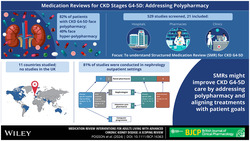

## 1 INTRODUCTION

In March 2017, the World Health Organization (WHO) issued an action plan, ‘Medication without Harm’, to deal with the global patient safety challenge posed by medication. Medication errors are inevitable and provoked in large part by fragile health systems. However, the challenge lies in reducing their frequency and impact. An action plan is a change programme aimed at improving and reducing risks. Polypharmacy, defined as the routine use of four or more regular medicines, was highlighted as an early ‘high priority’ area for action, with an aim to decrease the level of severe avoidable harm by 50% within 5 years.^1^

Polypharmacy is driven by an ageing and increasingly frail population living with multiple health conditions (comorbidities) and by the prescription of preventative medication for single‐organ diseases. Frailty describes the loss of in‐built reserves and vulnerability to adverse events.^2^

The adverse effects of polypharmacy include, but are not limited to, an increased risk of drug interactions and adverse drug events, with reduced quality of life (QoL) and adherence. Fifty percent of people prescribed five or more medicines are not taking them as intended. The Kings Fund defines polypharmacy as: ‘appropriate polypharmacy’, following medicines optimization; or ‘problematic polypharmacy’ when multiple medications are prescribed inappropriately, or where the intended benefit of the medication is not realized.^3^

High‐risk situations which can result in harm from medication may concern the person (elderly or with kidney disease), the medication (complex medication regimes) and the situation (transitions between care facilities).^1^ In patients with chronic kidney disease (CKD), polypharmacy is associated with a higher risk of all‐cause mortality, kidney failure, faster estimated glomerular filtration rate (eGFR) decline, lower QoL, adverse drug reactions, potentially inappropriate medications and higher medication non‐adherence.^4^ Once more than 15 different regular medicines are taken per day, this treatment burden significantly decreases QoL.^5^ Visiting many specialist prescribers, hyper‐polypharmacy, high‐risk medicines, risky combination of medicines and frailty are all common among people living with CKD stage G4‐5D.^6^ Polypharmacy rates imply that this population is not benefitting from medication optimization.^7^

People living with CKD stage G4‐5D also live with multiple other health conditions.^8^, ^9^ Co‐ordination of care between specialists is often disjointed.^6^, ^10^ Comorbidity, together with controlling progressive complications of kidney disease, leads to one of the highest medicine burdens of all populations: 82% experience polypharmacy and 40% experience hyperpolypharmacy (≥10 different medicines a day).^4^ The prevalence of polypharmacy increases as kidney function declines, peaking in patients receiving dialysis or kidney transplants.^4^, ^11^, ^12^

In response to the WHO, the United Kingdom Secretary of State for Health commissioned a short working group. They highlighted that concepts such as shared decision‐making and education are key areas to encourage, support and engage patients and families in guiding decisions regarding their medication.^13^

Following this, the National Institute for Health and Care Excellence (NICE) has developed clinical guidelines for multimorbidity, concurrent multiple diseases, and conditions in one person,^13^ medicine adherence, empowering medication taking,^14^ and medicines optimization to enable the best possible outcomes from medicines management.^15^ The support for the implementation of these standards for people living with CKD stage G4‐5D has focused on primary care services.

Comprehensive evidence‐based structured medication reviews (SMRs) can empower patients to understand the risks and benefits of medication through shared decision‐making. SMRs improve the quality of care, reduce harm and offer value for money by reducing adverse drug events, side effects, hospitalization and medicine waste.^16^ The medicines offered are aligned to realistic treatment goals in partnership with the patient.

While the use of SMR is outlined in patients with multiple morbidities in primary care, less is known about its use in patients with CKD who are in a kidney hospital environment. Therefore, it is necessary to understand how SMR interventions have been studied in this population, specifically, the medicines addressed, outcomes measured, facilitators and barriers to delivery and previously identified evidence gaps.

## 2 METHODS

### 2.1 Design and reporting

This scoping review was conducted in accordance with the JBI methodology for scoping reviews.^17^ The review was registered on the Open Science Framework (OSF.IO/HSW96; https://osf.io/hsw96/).^18^ The Preferred Reporting Items for Systematic Reviews and Meta‐Analyses extension for Scoping Reviews (PRISMA‐ScR) checklist was used.^19^ Details of protocol changes are presented in Appendix B.

### 2.2 Research questions and objectives

To understand the extent and nature of previous SMR interventions for people living with advanced CKD (stage G4‐5D), we developed four research questions regarding interventions and outcomes.

1. What SMR interventions have previously been tested within the CKD stage G4‐5D population?
  To describe and categorize SMR interventions, including the type of study design, target populations (e.g., stage of kidney disease), setting (i.e., primary care, acute hospital setting, outpatient clinic), categorize the types of interventions (e.g., medication review or targeted deprescribing), medication addressed, and details of the responsible professional (i.e., pharmacist or nephrologist).
2. What medicines were studied during SMR intervention for CKD stage G4‐5D?
  To identify and characterize medicines addressed into British National Formulary (BNF) classes.
3. What outcomes were measured during SMR interventions for CKD stage G4‐5D?
  To identify and categorize outcomes, including any clinical, patient‐important, medication‐related or experience‐related outcomes.
4. What research priorities have been identified for SMR for CKD stage G4‐5D?
  To describe identified research priorities for future research.

Our scoping review approach recognizes the value of this method for exploring the available literature and mapping the nature and type of available evidence. The search for evidence was directed specifically for those cared for by the kidney services in an acute hospital environment. This review included both quantitative and qualitative studies.

### 2.3 Search strategy

Potentially eligible studies were identified by searching the electronic databases MEDLINE, EMBASE, CINAHL and the Cochrane Central Register of Controlled Trials (CENTRAL) from database inception to 19 October 2023. An initial limited search of MEDLINE, EMBASE and CINAHL was performed to identify articles on this topic. The words contained in the titles and abstracts of the relevant articles and the index terms used to describe the articles were used to develop a full search strategy, as detailed in Appendix A. Our search strategy was based on the advice of experienced clinical librarians (R.H. and A.R.). There were no language or publication date restrictions, reflecting our interest in mapping all the research conducted to date.

#### 2.3.1 Search themes

We recognized that the term ‘medication review” was relatively new in the literature. To make our search more inclusive where this term was not index‐linked, we combined the following two searches.

- SMR in the context of CKD stage G4‐5D
- Polypharmacy and morbidity in the context of CKD stage G4‐5D.

### 2.4 Eligibility criteria

**Inclusion criteria:** A description of the inclusion criteria is provided in Table 1. The SPIDER (Sample, Phenomenon of Interest, Design, Evaluation, Research type) framework was used.

**TABLE 1:** SPIDER criteria.

Criterion	Description
**S**ample	Patients with CKD4‐5D: CKD is defined by KDIGO as abnormalities of kidney structure or function, present for 3 months, with implications for health. KDIGO staging of chronic kidney disease is based on GFR, stage G4 (severely decreased) GFR 15–29 mL/min/1.72 m^2^ stage, G5 (kidney failure) GFR ≤ 15 mL/min/1.72 m^2^, and includes those receiving dialysis (D).
**P**henomenon of **I**nterest	Any medication review intervention focused on addressing polypharmacy and any descriptions of complications.
**D**esign	All published study designs describing an intervention to address the management of polypharmacy; including but not limited to retrospective case reviews, retrospective observational studies, and case series including 10 or more participants.
**E**valuation	Descriptions of the population included will be examined for the following factors: age, degree of renal dysfunction and renal replacement therapies received. Descriptions of the types of interventions will be examined for the following factors: design (medication review or targeted deprescribing), setting (primary, secondary, older‐care, nephrology), follow‐up schedules, outcomes (patient‐related, medication‐related, qualitative), and the professional conducting the intervention. For medication: type and range according to BNF classification. Research gaps identified and description of call for future research.
**R**esearch type	All published research articles will be included.

Abbreviations: BNF, British National Formulary; CKD, chronic kidney disease; GFR, glomerular filtration rate.

**Exclusion criteria:** Research papers that included patients on the transplant waiting list, those with a limited life expectancy due to other life‐limiting illnesses, those with fewer than 10 participants, or those with acute kidney injury were excluded.

### 2.5 Screening and data extraction

Two review authors (C.P. and R.C.A.) independently screened the titles and abstracts of the identified studies and the full texts of the potentially eligible studies. Duplicates were automatically removed using Endnote and Covidence software. Disagreements regarding the eligible studies were resolved through discussion.

One reviewer (C.P.) extracted data from the eligible studies into a pilot data extraction template (Appendix D) as follows:

- *Study‐level data*: authors, title, year of publication (based on online publication), country, study characteristics (randomized controlled trial [RCT], cohort, case‐controlled, single or multicentre), setting (primary, nephrology), number of participants, type of medication review intervention (all prescribed medicines, of targeted medicines), intervention design and aim (including medication class and descriptors of the target population).
- *Outcome‐level data*: clinical (e.g., reduced admission to hospital), patient‐important (e.g., living with medicines visual analogue score) and quality of life (e.g., EQ‐5D‐5L scores), medication‐related (medication or pill counts), experience‐related (i.e., patient and clinician perspectives) and knowledge gaps were identified for further research.

The structured Excel template recorded data at both study and outcome levels. R.C.A., who was blinded, checked 10% of all data extracted for accuracy. Only minor discrepancies were found; therefore, no further accuracy checks were performed. We did not formally assess the quality of the included studies. Consensus on eligible studies was reached.

### 2.6 Data synthesis and analysis

Using the intervention description, we further classified interventions to describe the target medicines against BNF categorization and against those highlighted as ‘problematic polypharmacy’ in the literature.

To organize the outcomes measures, we categorized them into:

- Clinical – complications of CKD, adverse events, hospitalization or mortality, blood pressure or changes in kidney function.
- Patient‐important – symptom control, QoL, medication knowledge or adherence.
- Medication‐related – medication counts, drug‐interactions, contraindications.
- Experience‐related – clinician and patient perspectives about the SMR process.

The results are summarized and presented according to the study design and hierarchy of evidence (Appendix E).

### 2.7 Patient and public involvement

The results of our review were shared with people living with CKD stage G4‐5D and their caregivers. We sought feedback on the utility and feasibility of medication reviews and compared our review findings to their lived experiences.

### 2.8 Nomenclature of targets and ligands

Key protein targets and ligands in this article are hyperlinked to corresponding entries in http://www.guidetopharmacology.org, and are permanently archived in the Concise Guide to PHARMACOLOGY 2023/24.^20^, ^21^, ^22^

## 3 RESULTS

**FIGURE 1:**
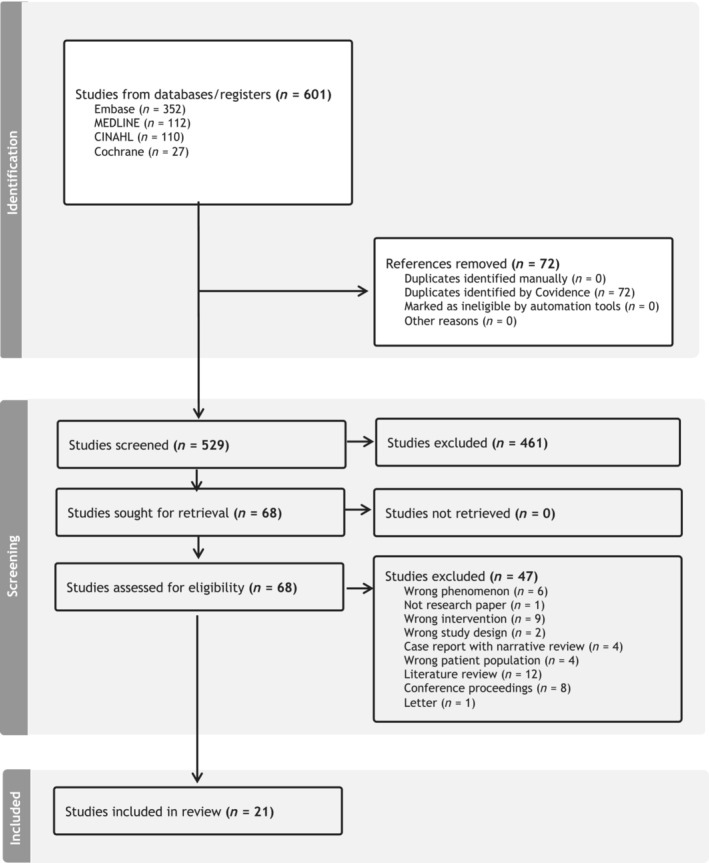
PRISMA‐ScR flow diagram illustrating the study identification, screening and inclusion process.

Of the 529 titles reviewed, 21 were included in this study. The references for all included studies are listed in Appendix C. The PRISMA‐SR flowchart is presented in Figure 1.

### 3.1 Characterisation of included studies

#### 3.1.1 Year of publication

The oldest study identified was published in 1997,^23^ but the majority (*n* = 12) were published since 2019.^24^, ^25^, ^26^, ^27^, ^28^, ^29^, ^30^, ^31^, ^32^, ^33^, ^34^, ^35^

#### 3.1.2 Country

**FIGURE 2:**
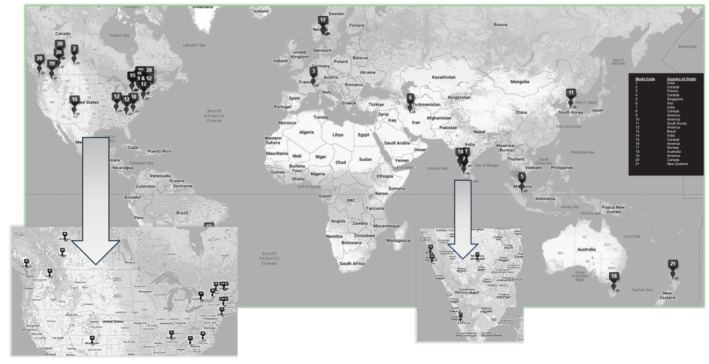
A global map of medication review studies for people living with CKD stage G4‐5D.

Studies were conducted in 11 countries: Canada (*n* = 5),^25^, ^26^, ^28^, ^35^, ^36^ United States (*n* = 5),^23^, ^29^, ^31^, ^37^, ^43^ India (*n* = 3),^24^, ^27^, ^38^ Australia,^34^ Brazil,^32^ France,^39^ Iran,^40^ Norway,^33^ New Zealand,^41^ Singapore^42^ and South Korea^30^ each with a single study identified. To date, no such studies have been conducted in multiple countries, and no such study has been conducted in the United Kingdom (Figure 2).

#### 3.1.3 Participants per country

Participants per country are shown in Table 2. The country with the greatest number of patients was the United States (*n* = 1042),^23^, ^29^, ^31^, ^37^, ^43^ followed by India (*n* = 441)^24^, ^27^, ^38^ and Singapore (*n* = 324).^42^ A total of 2888 people living with CKD stages G1‐5D were included in the studies (ranging from 10 to 726 participants/study), with qualitative studies having the lowest number of participants.

**TABLE 2:** Number of participants and type of intervention per country.

Country of origin	Study number	Number of participants	Structured medication review	Targeted deprescribing	Number of studies
USA^23^, ^29^, ^31^, ^37^, ^43^	9, 10, 12, 16, 19	1042	4 (9, 12, 16, 19)	1 (10)	5
India^24^, ^27^, ^38^	1, 7, 14	441	2 (1, 7)	1 (14)	3
Singapore^42^	5	324	1		1
Canada^25^, ^26^, ^28^, ^35^, ^36^	2, 4, 8, 15, 20	279	1 (2)	4 (4, 8, 15, 20)	5
Australia^34^	18	204	1		1
Norway^33^	17	180	1		1
Brazil^32^	13	100		1	1
South Korea^30^	11	95	1		1
Iran^40^	6	92	1		1
France^39^	3	67	1		1
New Zealand^41^	21	64	1		1
**Total**	**21**	**2888**	**14**	**7**	**21**

Full details of the year of publication, country, study design and distribution of sample sizes are summarized in Appendix E.

#### 3.1.4 Participants by stage of kidney function and age

Participation by stage of kidney function and age are shown in Table 4. Twelve (57%) studies focused solely on people receiving haemodialysis.^23^, ^26^, ^27^, ^28^, ^29^, ^35^, ^36^, ^37^, ^38^, ^40^, ^41^, ^42^ Five studies (24%) included only adults aged 65 years or older.^26^, ^30^, ^33^, ^34^, ^35^ One study (5%) had an upper age limit of 75 years,^38^ while another study had an upper age limit of 90 years.^40^ Two studies included people receiving conservative care and those who decided not to receive dialysis.^32^, ^33^

#### 3.1.5 Study design

The study design is shown in Table 3. Study designs included RCTs (*n* = 5),^33^, ^37^, ^38^, ^43^ including one retrospective secondary analysis of a larger trial,^35^ cohort studies (*n* = 3) (two retrospective,^31^, ^42^ one prospective^40^), observational case series (*n* = 10) (seven prospective,^23^, ^24^, ^27^, ^28^, ^36^, ^39^, ^41^ three retrospective^30^, ^32^, ^34^) and qualitative studies (*n* = 3) (one descriptive phenomenological study,^25^ one grounded theory analysis,^26^ and one rapid‐thematic analysis^29^) (Appendix E).

**TABLE 3:** Study design, style, intervention and follow‐up periods.

Study	Study number	Style	Structured medication review	Targeted deprescribing	Follow‐up						Total
					None	1‐month	3‐month	4‐month	6‐month	12‐month	
Randomized controlled trial^33^, ^35^, ^37^, ^38^, ^43^	14, 16, 17, 19	Prospective	3 (16, 17, 19)	1 (14)			1 (19)		2 (16, 17)	1 (14)	4
	20	Secondary analysis		1		1					1
Observational cohort study^31^, ^40^, ^42^	6	Prospective	1						1		1
	5, 12	Retrospective	2			1 (5)			1 (12)		2
Observational case series^23^, ^24^, ^27^, ^28^, ^30^, ^32^, ^34^, ^36^, ^39^, ^41^	1, 3, 7, 8, 9, 15, 21	Prospective	5 (1, 3, 7, 9, 21)	2 (8,15)	3 (1, 9, 21)			1 (8)	3 (3, 7, 15)		7
	11, 13, 18	Retrospective	2 (11, 18)	1 (13)	1 (18)		1 (11)		1 (13)		3
Qualitative study^25^, ^26^, ^29^	2, 4, 10		1 (2)	2 (4, 10)							3

#### 3.1.6 Setting

A description of the setting is shown in Table 4. Seventeen studies (81%) were undertaken within nephrology outpatient settings,^23^, ^26^, ^27^, ^28^, ^29^, ^30^, ^31^, ^32^, ^33^, ^36^, ^37^, ^38^, ^39^, ^40^, ^41^, ^42^, ^43^ 12 (57%) within dialysis units,^23^, ^26^, ^27^, ^28^, ^29^, ^31^, ^36^, ^37^, ^38^, ^40^, ^41^, ^42^ 11 (52%) enrolled only patients undergoing haemodialysis,^23^, ^26^, ^27^, ^28^, ^29^, ^36^, ^37^, ^38^, ^40^, ^41^, ^42^ and three studies (14%) included peritoneal dialysis and haemodialysis.^33^, ^34^, ^39^ Of the remaining four studies, three were completed during an acute hospital stay^24^, ^34^, ^35^ and one explored SMRs in a community pharmacy setting.^25^

**TABLE 4:** Participants by stage of chronic kidney disease intervention age and description of the setting.

Renal function	Study number	Structured medication review	Targeted deprescribing	Age of participants	Description of setting	Detail description of setting
CKD Stage G1‐G5 including transplant^25^	2	1		≥ 18 yrs old	Community Setting	Community pharmacy ‐ Nephrology patients
CKD Stage G1‐G5^39^	3	1		≥ 18 yrs old	Nephrology	Out‐patient nephrology
CKD Stage G1‐G5 conservative care only^32^	13		1	≥ 18 yrs old	Nephrology	Out‐patient nephrology clinic
CKD Stage G3‐G5^34^	18	1		≥ 65 yrs old	Acute hospital	In‐patient
CKD Stage G3‐G5^43^	19	1		≥ 18 yrs old	Nephrology	Out‐patient clinic following hospitalization
CKD Stage G3‐G5D^24^	1	1		≥ 18 yrs old	Acute hospital ‐ Intensive care	In‐patient
CKD Stage G3‐G5D^30^	11	1		≥ 65 yrs old	Nephrology	Out‐patient ambulatory care clinic
CKD Stage G5‐G5D (conservative, PD & HD)^33^	17	1		≥ 65 yrs old	Nephrology	Out‐patient clinic
CKD Stage G5D (PD & HD)^31^	12	1		≥ 18 yrs old	Nephrology	Out‐patient dialysis clinic
CKD Stage G5D‐HD^26^	4		1	≥ 65 yrs old	Nephrology	Out‐patient haemodialysis
CKD Stage G5D‐HD^35^	20		1	≥ 65 yrs old	Acute hospital	In‐patient
CKD Stage G5D‐HD^23^, ^27^, ^28^, ^36^, ^37^, ^41^, ^42^	5, 7, 8, 9, 15, 16, 21	5 (5, 7, 9, 16, 21)	2 (8, 15)	≥ 18 yrs old	Nephrology	Out‐patient haemodialysis
CKD Stage G5D‐HD^29^	10		1	≥ 18 yrs old	Nephrology	Out‐patient haemodialysis following hospitalization
CKD Stage G5D‐HD^38^	14		1	18–75 yrs old	Nephrology	Out‐patient haemodialysis
CKD Stage G5D‐HD^40^	6	1		18–90 yrs old	Nephrology	Out‐patient haemodialysis

#### 3.1.7 Follow‐up

Follow‐up is shown in Table 3. Twelve (57%) of the studies were prospective interventions,^23^, ^24^, ^27^, ^28^, ^33^, ^36^, ^37^, ^38^, ^39^, ^40^, ^41^, ^43^ of which four were RCTs,^33^, ^37^, ^38^, ^43^ one was a cohort study^40^ and seven (33%) were observational case series.^23^, ^24^, ^27^, ^28^, ^36^, ^39^, ^41^ Six (29%) of the studies were retrospective interventions,^30^, ^31^, ^32^, ^34^, ^35^, ^42^ of which two were cohort studies,^31^, ^42^ three (14%) were observational case series^30^, ^32^, ^34^ and one was a secondary analysis of an RCT.^35^ The follow‐up for all intervention studies^23^, ^24^, ^27^, ^28^, ^30^, ^31^, ^32^, ^33^, ^34^, ^35^, ^36^, ^37^, ^38^, ^39^, ^40^, ^41^, ^42^, ^43^ varied from none to 12 months (mode 6 months).

### 3.2 Classification of intervention

#### 3.2.1 Interventions by design

Interventions per design are shown in Table 3. Interventions varied in style; two‐thirds (*n* = 14),^23^, ^24^, ^25^, ^27^, ^30^, ^31^, ^33^, ^34^, ^37^, ^39^, ^40^, ^41^, ^42^, ^43^ of all studies used SMRs, optimizing all medicines prescribed. The remaining third (*n* = 7),^26^, ^28^, ^29^, ^32^, ^35^, ^36^, ^38^ were deprescribing studies limited to medicines considered appropriate for deprescribing. In the United States, most studies were SMRs, while in Canada, most studies identified targeted deprescribing interventions. For the 12 studies in the haemodialysis population, six (29%) were SMR interventions,^23^, ^27^, ^37^, ^40^, ^41^, ^42^ and six (29%) were targeted deprescribing intervention.^26^, ^28^, ^29^, ^35^, ^36^, ^38^

#### 3.2.2 Intervention by purpose

**FIGURE 3:**
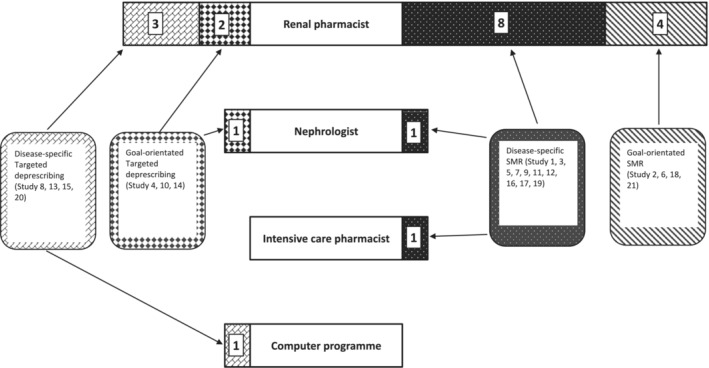
A butterfly chart showing the medication review studies by their aim, style and the professional undertaking them.

Interventions by purpose are shown in Figure 3. Fourteen studies (67%) focused the intervention upon disease‐specific outcomes.^23^, ^24^, ^27^, ^28^, ^30^, ^31^, ^32^, ^33^, ^35^, ^36^, ^37^, ^39^, ^42^, ^43^ The other seven studies (33%),^25^, ^26^, ^29^, ^34^, ^38^, ^40^, ^41^ designed their intervention around goal‐orientated outcomes, evaluating medication regime around the individual's priorities.

#### 3.2.3 Intervention by setting

Intervention by setting is shown in Table 4. Seventeen studies (81%) were undertaken within nephrology outpatient settings,^23^, ^26^, ^27^, ^28^, ^29^, ^30^, ^31^, ^32^, ^33^, ^36^, ^37^, ^38^, ^39^, ^40^, ^41^, ^42^, ^43^ 12 (57%) within dialysis units,^23^, ^26^, ^27^, ^28^, ^29^, ^31^, ^36^, ^37^, ^38^, ^40^, ^41^, ^42^ 11 (52%) enrolled only patients undergoing haemodialysis,^23^, ^26^, ^27^, ^28^, ^29^, ^36^, ^37^, ^38^, ^40^, ^41^, ^42^ and three studies included peritoneal dialysis and haemodialysis.^30^, ^31^, ^33^ Of the remaining four studies, three were completed during an acute hospital stay^24^, ^34^, ^35^ and one explored SMRs in a community pharmacy setting.^25^

#### 3.2.4 Interventions by medicine category

A description of the medicines included in the studies is included in Table 5. Fifteen studies (71%) described the medications included in their investigation.^24^, ^27^, ^28^, ^29^, ^30^, ^31^, ^32^, ^33^, ^34^, ^35^, ^36^, ^37^, ^39^, ^41^, ^43^ Six studies were not included in this chart as they did not include specific descriptions of medications, including four (22%) quantitative studies (one randomized controlled trial,^38^ two cohort studies,^40^, ^42^ and one case series^23^) and two (67%) qualitative studies.^25^, ^26^ Medications were mapped by BNF class.

**TABLE 5:** Classification of medicines included in studies.

Study number	1 SMR	3 SMR	7 TD	8 TD	10 TD	11 SMR	12 SMR	13 TD	15 TD	16 SMR	17 SMR	18 SMR	19 SMR	20 TD	21 SMR	Medicines included
**Digestive tract**																
PPI		✓	✓	✓		✓	✓		✓	✓	✓	✓	✓	✓		11
H2 receptor antagonist		✓	✓				✓	✓		✓		✓				6
Anti‐emetics		✓	✓			✓	✓			✓	✓					6
Prokinetic agents		✓				✓	✓			✓	✓					5
Laxatives		✓				✓	✓	✓		✓	✓			✓		7
**Cardiovascular system**																
Digoxin	✓					✓	✓			✓	✓	✓				6
Amiodarone	✓					✓	✓			✓	✓	✓				6
ACE‐I or ARB		✓	✓			✓	✓	✓		✓	✓	✓	✓	✓	✓	11
β‐blocker	✓	✓	✓			✓	✓	✓		✓	✓	✓	✓	✓	✓	12
Aldosterone antagonists						✓					✓	✓			✓	4
Calcium antagonist	✓	✓	✓			✓	✓	✓		✓	✓	✓	✓	✓	✓	12
Centrally acting antihypertensive	✓				✓	✓	✓	✓		✓	✓	✓	✓	✓		10
Potassium channel activators											✓					1
Nitrates	✓						✓			✓		✓		✓		5
Vasodilators							✓			✓						2
Fibrate								✓		✓		✓				3
Statins			✓	✓		✓	✓		✓	✓	✓	✓	✓	✓	✓	11
Antiplatelets			✓	✓		✓	✓			✓	✓	✓	✓	✓	✓	10
Anticoagulants						✓	✓			✓	✓	✓		✓		6
Combined Antiplatelets +/ ‐ anticoagulants						✓						✓		✓		3
Diuretics	✓	✓	✓	✓		✓	✓		✓	✓	✓	✓	✓	✓		12
**Respiratory system**																
Systemic corticosteroids						✓	✓			✓	✓				✓	5
Inhaled β2 agonist/antimuscarinics	✓		✓	✓		✓	✓			✓	✓				✓	5
Sedating antihistamines						✓	✓			✓	✓					4
**Central nervous system**																
Anticholinergics					✓	✓	✓			✓	✓	✓				6
Benzodiazepines, Z‐Drugs		✓		✓	✓	✓	✓			✓	✓	✓	✓	✓		10
Antipsychotics					✓	✓					✓		✓	✓		5
Antidepressants						✓	✓			✓	✓	✓	✓	✓		7
Tramadol												✓	✓			2
Opioid analgesics					✓	✓	✓	✓		✓	✓		✓	✓	✓	9
Gabapentinoids								✓				✓	✓	✓		4
Nicotine replacement								✓								1
**Infections**																
Antibiotics	✓						✓	✓		✓			✓	✓	✓	7
Antifungals								✓								1
Antivirals								✓								1
Vaccines							✓									1
**Endocrine**																
Oral hypoglycaemics		✓	✓	✓		✓	✓	✓		✓	✓	✓	✓	✓	✓	12
Insulin	✓	✓					✓			✓			✓	✓	✓	7
Tolvaptan	✓															1
**Urinary tract disorders**																
α1‐blockers		✓	✓	✓	✓	✓	✓	✓	✓	✓	✓	✓	✓			12
Anti‐androgens		✓					✓			✓	✓					4
Phosphodiesterase inhibitor	✓					✓	✓				✓					4
**Anaemia**																
Erythropoietin		✓					✓								✓	3
Iron		✓	✓				✓	✓				✓		✓	✓	7
**CKD‐MBD**																
Vitamin D	✓	✓	✓				✓	✓		✓	✓		✓	✓	✓	10
Oral calcium	✓	✓	✓					✓		✓		✓	✓		✓	8
Phosphate binders		✓	✓					✓		✓			✓			5
Calcimimetics		✓	✓				✓			✓			✓			5
Bisphosphonates								✓			✓					2
**Electrolyte disturbances**																
Potassium binders								✓								1
Sodium bicarbonate			✓													1
**MSK and Joint treatments**																
NSAIDs		✓				✓	✓	✓			✓					5
Corticosteroids											✓	✓				2
Colchicine						✓		✓		✓	✓				✓	4
Allopurinol or febuxostat			✓	✓		✓	✓	✓		✓	✓	✓		✓	✓	10
Quinine									✓		✓					2
Muscle Relaxants (baclofen)					✓											1
Skin																
Dermatological agents		✓					✓			✓						3
**No. of BNF classes included per study**	**14**	**22**	**18**	**8**	**7**	**29**	**38**	**24**	**5**	**37**	**35**	**27**	**21**	**23**	**18**	

Abbreviations: ACE‐I, angiotensin‐converting enzyme inhibitors; ARB, angiotensin II receptor blockers; BNF, British National Formulary; CKD‐MBD, Chronic Kidney Disease‐Mineral Bone Disorder; MSK, musculoskeletal; NSAIDs, non‐steroidal anti‐inflammatory drugs; PPI, proton pump inhibitors; SMR, structured medication review; TD, targeted deprescribing.

Proton pump inhibitors,^27^, ^28^, ^30^, ^31^, ^33^, ^34^, ^35^, ^36^, ^37^, ^39^, ^43^ angiotensin‐converting enzyme inhibitors or angiotensin‐II receptor blockers,^27^, ^30^, ^31^, ^32^, ^33^, ^34^, ^35^, ^37^, ^39^, ^41^, ^43^ beta‐blockers,^24^, ^27^, ^30^, ^31^, ^32^, ^33^, ^34^, ^35^, ^37^, ^39^, ^41^, ^43^ calcium antagonists,^24^, ^27^, ^30^, ^31^, ^32^, ^33^, ^34^, ^35^, ^37^, ^39^, ^41^, ^43^ centrally acting antihypertensives,^24^, ^29^, ^30^, ^31^, ^32^, ^33^, ^34^, ^35^, ^37^, ^43^ alpha‐blockers,^27^, ^28^, ^29^, ^30^, ^31^, ^32^, ^33^, ^34^, ^36^, ^37^, ^39^, ^43^ statins,^27^, ^28^, ^30^, ^31^, ^33^, ^34^, ^35^, ^36^, ^37^, ^41^, ^43^ anti‐platelets,^27^, ^28^, ^30^, ^31^, ^33^, ^34^, ^35^, ^37^, ^41^, ^43^ diuretics,^24^, ^27^, ^28^, ^30^, ^31^, ^33^, ^34^, ^35^, ^36^, ^37^, ^39^, ^43^ benzodiazepines or Z‐drugs,^28^, ^29^, ^30^, ^31^, ^33^, ^34^, ^35^, ^37^, ^39^, ^43^ oral hypoglycaemic agents,^27^, ^28^, ^30^, ^31^, ^32^, ^33^, ^34^, ^35^, ^37^, ^39^, ^41^, ^43^ vitamin D,^24^, ^27^, ^31^, ^32^, ^33^, ^35^, ^37^, ^39^, ^41^, ^43^ urate‐lowering therapy (allopurinol or febuxostat)^27^, ^28^, ^30^, ^31^, ^32^, ^33^, ^34^, ^35^, ^37^, ^41^ were all included individually in 10 or more of the studies.

#### 3.2.5 Delivery of intervention by profession

A description of the profession delivering the intervention is included in Table 6. Seventeen (81%) studies were conducted by a renal pharmacist,^23^, ^25^, ^26^, ^27^, ^28^, ^29^, ^30^, ^31^, ^32^, ^34^, ^36^, ^37^, ^38^, ^39^, ^40^, ^41^, ^42^, ^43^ one study was conducted by an intensive care pharmacist.^24^ Fifteen (83%) quantitative intervention studies, 11 SMRs^23^, ^27^, ^30^, ^31^, ^34^, ^37^, ^39^, ^40^, ^41^, ^42^, ^43^ and four targeted deprescribing studies^28^, ^32^, ^36^, ^38^ were undertaken by a renal pharmacist.

**TABLE 6:** Intervention by profession.

Professional	Study number	Quantitative		Qualitative		Total
		Structured medication review	Targeted deprescribing	Structured medication review	Targeted deprescribing	
Renal pharmacist^23^, ^25^, ^26^, ^27^, ^28^, ^30^, ^31^, ^32^, ^34^, ^36^, ^37^, ^39^, ^40^, ^41^, ^42^, ^43^	2, 3, 4, 5, 6, 7, 8, 9, 11, 12, 13, 14, 15, 16, 18, 19, 21	11 (3, 5, 6, 7, 9, 11, 12, 16, 18, 19, 21)	4 (8, 13, 14, 15)	1 (2)	1 (4)	17
Intensive care pharmacist^24^	1	1				1
Nephrologist^29^, ^33^	10, 17	1 (17)			1 (10)	2
Computer program^35^	20		1			1
**Total**		**13**	**5**	**1**	**2**	**21**

### 3.3 Outcome measurements

**FIGURE 4:**
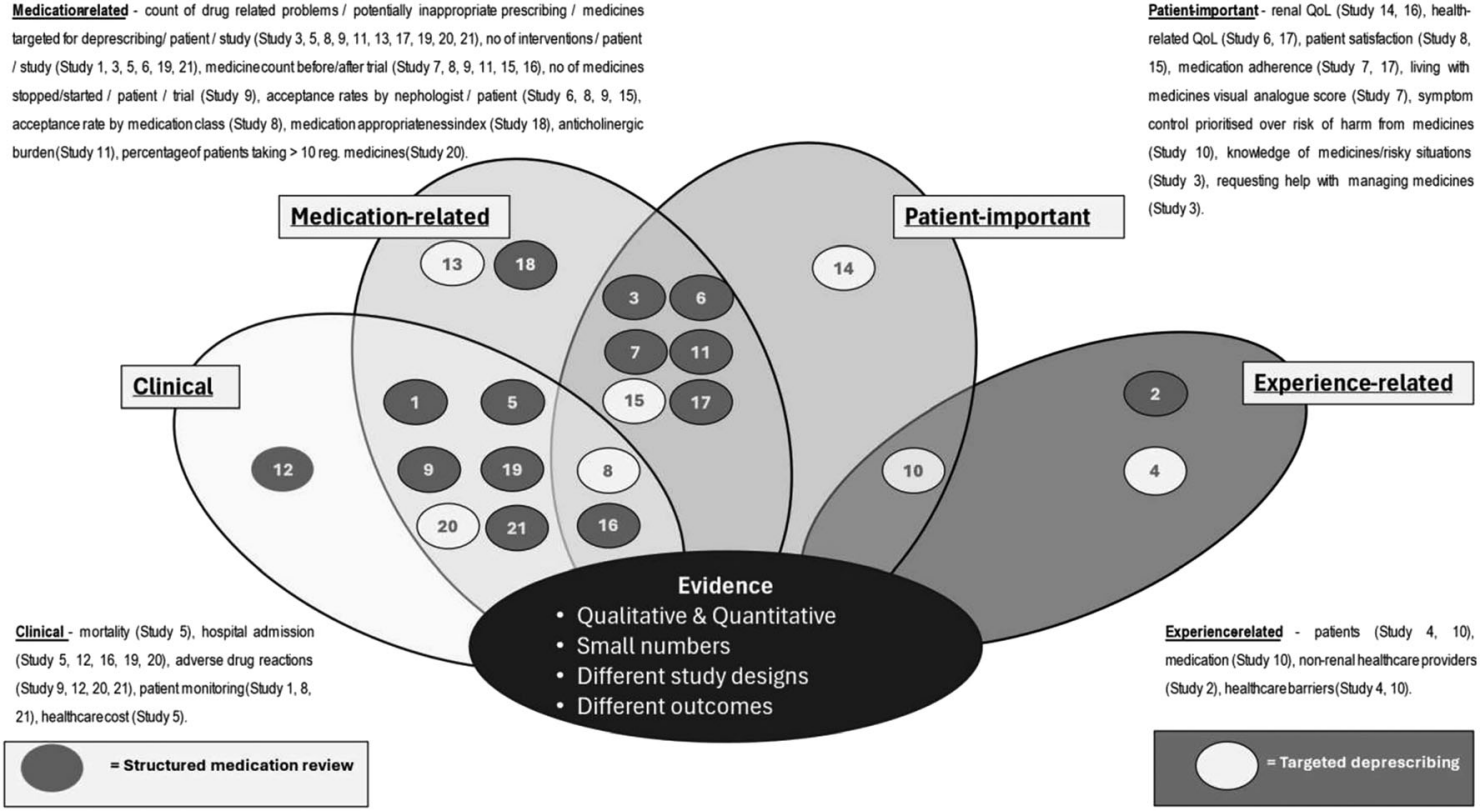
Venn diagram of outcomes measured in medication review studies for CKD stage G4‐5D patients.

Outcome measurements, summarised in Figure 4 and Appendix F, were categorized into:

- Clinical
- Medication‐related
- Patient‐important
- Experience‐related

These outcomes were classified according to the study type. Most clinical, patient‐important and medication‐related outcomes have been reported in quantitative studies.^23^, ^24^, ^27^, ^28^, ^30^, ^31^, ^32^, ^33^, ^34^, ^35^, ^36^, ^37^, ^38^, ^39^, ^40^, ^41^, ^42^, ^43^ Hall et al. identified one important patient outcome theme from a qualitative study in which 10 patients prioritized symptom control over any risk of harm from medicines.^29^ All the experience‐related outcomes were reported from the qualitative studies.^25^, ^26^, ^29^

#### 3.3.1 Clinical outcome themes

Four clinical outcome themes were identified from nine (50%) quantitative studies^23^, ^24^, ^28^, ^31^, ^35^, ^37^, ^41^, ^42^, ^43^: rates of hospitalization (including 30‐day readmission rates), duration of hospital stay, mortality and adverse effects. These were revealed in seven of the 14 SMR intervention studies^23^, ^24^, ^31^, ^37^, ^41^, ^42^, ^43^ and two of the seven targeted deprescribing studies.^28^, ^35^

Large SMR studies from the United States found correlations with reductions in all‐cause hospitalization, 30‐day readmission rates and hospital stay if hospitalized, with no effect on mortality.^31^, ^37^ This association was not replicated by Tuttle et al.^43^

Four studies (three SMRs^23^, ^31^, ^43^ and one targeted deprescribing)^35^ identified adverse drug reactions. Gerardi et al., a targeted deprescribing study,^28^ focused on proton pump inhibitors with no indication. This approach was considered unsuccessful. Proton pump inhibitors had to be reintroduced in 62.5% (five out of eight) patients for whom they were deprescribed. In patients undergoing dialysis, high gastrointestinal bleeding rates have been reported.^35^ They concluded that deprescribing proton pump inhibitors in patients with CKD stage G4‐5D with no such indication may be associated with harm.

#### 3.3.2 Medication‐related outcome themes

Nine medication‐related outcome themes were identified from 16 (89%) quantitative studies (11 SMRs,^23^, ^24^, ^30^, ^33^, ^34^, ^37^, ^39^, ^40^, ^41^, ^42^, ^43^ and five targeted deprescribing studies^27^, ^28^, ^32^, ^35^, ^36^).

SMR studies reported rates of identification of medication‐related problems and the prevalence of inappropriate medication use.^23^, ^30^, ^33^, ^35^, ^39^, ^41^, ^42^, ^43^ These medication‐related problems include drug–drug or drug–food interactions, side‐effects, dose optimization due to kidney function, contraindications, patient adherence, adherence to guidelines, supply‐chain problems and communication between healthcare providers.^23^, ^30^, ^33^, ^35^, ^39^, ^41^, ^42^, ^43^

Targeted deprescribing studies reported the total number of medicines prescribed against target numbers of medicines deprescribed and successful deprescribing rates.^28^, ^32^ Six of the SMR studies recorded the number of interventions per patient and/or study.^24^, ^39^, ^40^, ^41^, ^42^, ^43^ Another six quantitative studies recorded medicine count before and after intervention,^23^, ^27^, ^28^, ^30^, ^36^, ^37^ and the number of medicines stopped and/or started per patient and per study.^23^ In studies involving renal pharmacists performing the intervention, acceptance rates of suggested medication changes were also recorded as acceptance rates by nephrologists and/or patients^23^, ^28^, ^36^, ^40^ and by medication classification.^28^ The complexity of medication regimes was measured by a medication appropriateness index^34^ an anticholinergic burden score^30^ and hyperpolypharmacy percentage.^35^

#### 3.3.3 Patient‐important outcomes themes

Seven patient‐important outcome themes were identified from nine (50%) quantitative studies, five SMRs^33^, ^37^, ^38^, ^39^, ^40^ and four targeted deprescribing studies.^27^, ^28^, ^29^, ^36^ The outcomes were: effect of intervention upon QoL^33^, ^37^, ^38^, ^40^ (specifically renal‐ and health‐related), medication adherence,^27^, ^33^ patient satisfaction,^28^, ^36^ living with medicines visual analogue score,^27^ prioritizing symptom control over potential risk of harm from medicines,^29^ describing a lack of knowledge of medicines/risky situations, and requesting support with daily management of medicines.^39^

Belaiche et al. found in their intervention study that over 80% of patients were unaware of the beneficial impact of their treatment, 85% were not aware of medical risk situations, 68% declared self‐medication habits (including NSAIDs) and over 30% requested help with medicines management.^39^

#### 3.3.4 Experience‐related outcome themes

Experience‐related themes were identified from the three qualitative studies.^25^, ^26^, ^29^ These were sub‐categorized as clinical,^25^, ^26^, ^29^ patient‐important^25^, ^26^, ^29^ and medication‐related.^26^, ^29^

Clinical themes related to the experience of SMRs or targeted deprescribing interventions were identified in all three studies. Kidney pharmacists and doctors voiced concern about non‐specialist pharmacists offering inappropriate recommendations during a community‐led medication review.^25^ Some community pharmacists expressed concern over managing complex patients with CKD stage G4‐5D, explaining that training was inadequate.^25^ Kidney doctors also expressed concern over unclear roles and undefined co‐management by multiple clinicians with time constraints and competing priorities.^29^ They also identified the barriers between healthcare settings, exacerbated by limited computer interoperability.^29^ Healthcare workers should increase communication between teams to improve trust.^25^ Concern was expressed over the sustainability of deprescribing clinics even though the intervention was considered successful.^26^

Patient‐important themes were also identified in all three papers.^25^, ^26^, ^29^ Hall et al. reported that patients prioritize symptom control over potential harm from medication, preferring to stop medication which is causing side‐effects or threatening independence.^29^ Patients liked the opportunity to discuss their medication^25^; however, they also expressed ambivalence and limited knowledge regarding the risk–benefit analysis.^26^, ^29^ Patients reported empowerment after gaining an understanding of the importance of medication, even if no drug was deprescribed.^26^

Medication‐related themes were identified in two studies.^26^, ^29^ Kidney doctors and patients expressed limited awareness of medicines deemed potentially inappropriate.^29^ Deprescribing was identified by patients as an opportunity to learn about their medication.^26^

### 3.4 Reported literature gaps

This scoping review reveals many calls for further research to understand how SMRs can optimize outcomes in patients with CKD stage G4‐5D. Deprescribing proton pump inhibitors for these patients when there is no pre‐determined indication is time‐consuming and may be associated with harm. Further research is required before this practice can be adopted.^28^

Eleven papers identified the need for further quantitative research to understand the true difference in outcomes of medication reviews.^24^, ^27^, ^30^, ^31^, ^34^, ^35^, ^36^, ^39^, ^41^, ^42^, ^43^ Many papers called for further research to be designed to produce high‐level evidence. This research needs to be adequately powered and of high quality, suggesting the need for multi‐centre RCTs^27^, ^31^, ^36^, ^41^, ^42^ with a long follow‐up.^24^, ^30^, ^35^ There is a gap in understanding what is the optimal point to deliver the medication review in order to achieve the greatest impact on clinical and patient‐important outcomes, specifically QoL, hospital admissions and medication adherence.^28^, ^33^, ^34^, ^35^, ^36^, ^40^, ^41^, ^43^ Future research should include a cost–benefit analysis, as rationalizing medicines may save costs.^31^, ^39^ There is also a need to determine the standards for an optimal SMR to provide consistent transferable care.^33^, ^37^

Two papers identified a need for further qualitative research to understand the behavioural changes required for adopting adequate measures and to design SMRs around the aspects that matter most to patients and carers.^25^, ^29^

## 4 DISCUSSION

### 4.1 Summary of findings

This scoping review provides a summary of the current evidence supporting SMRs for patients with CKD stage G4‐5D in outpatient nephrology, acute hospital admission and community pharmacy settings across 11 countries. Only a few studies (*n* = 21) were identified. In these studies, SMR as an intervention showed promise as a strategy to reduce the inappropriate practice of polypharmacy for adults living with CKD stage G4‐5D.

More than 50% of studies focused on haemodialysis populations. Different kidney centres approach polypharmacy differently. In Canada, the focus has been on targeted deprescribing of pre‐determined medicines. Other countries have taken a more patient‐centred focus, highlighting omitted and unnecessary medicines through the SMR. The latter approach is recommended by the United Kingdom's NICE. The NICE recommends that SMR interventions should be co‐developed by patients and clinicians using shared decision‐making principles.^16^, ^44^

Two‐thirds of the studies designed their intervention around optimizing disease‐specific outcomes. The remaining third of the studies adopted a person‐centred approach by focusing the intervention upon goal‐oriented outcomes. This style enables the medication offered to be personalized and align with an individual's goals for treatment. It facilitates engagement, shared‐decision making and leads to positive outcomes as perceived by patients.^47^, ^48^

The wide range of medicines (14 BNF classes or more) included in 12 intervention studies describe the medicines on offer to patients with CKD stage G4‐5D. These complex regimes must be assessed to determine whether the harms outweigh the benefits. Bleeding, hypoglycaemic hypotension, cognitive impairment and cardiovascular (QT‐prolonging) combinations are medicine‐related risks. The SMR in this population was more complex than that in populations with other comorbidities.

Most identified medication review interventions (*n* = 18) were conducted by renal pharmacists. These findings indicate that although nephrologists supervise prescribing and determine the treatment goals for people with CKD stage G4‐5D, their focus may not be on the specifics required by medication reviews to support the practicalities of medication taking. SMR requires an interdisciplinary team approach for successful delivery.^16^

To assess the value of medication, we need to assess its impact on the lives of the people who take it. QoL, as an outcome, was reported in only four (22%) of the identified medication review interventions.^33^, ^37^, ^38^, ^40^ The current small number of trials and their design means that the impact of SMR interventions on QoL in the population with CKD stage G4‐5D is yet to be understood. SMR may improve QoL by facilitating and supporting patient‐specific complexities in medical management at home. Since a medication review intervention assesses the value of medication to an individual, QoL outcomes should be included in future research.

### 4.2 Comparison with the literature

Medication review has been widely described as an important intervention since the late 1990s when the term was first conceptualized.^49^ It has been conducted in many ways; from an opportunistic review during a prescriber–patient consultation to a structured review with the patient (and their family members or carers where appropriate) together with their full medical records. This structured approach has been shown to improve safety, efficacy and adherence to medication. Lias et al. harmonized the definition of medication review through an international Delphi consensus survey. This study describes the detailed steps for inclusion to facilitate the prevention, assessment and follow‐up of patients and their medication‐related problems.^50^

Other recent studies have further refined the classification into disease‐orientated or goal‐orientated.^47^, ^48^ Disease‐orientated medication reviews focus upon disease‐specific outcomes such as controlling blood pressure to reduce the risk of kidney failure or controlling lipids to reduce the incidence of cardiovascular disease. They focus on the optimal prescription associated with the specific comorbidities the patient has.^51^ This approach may overlook the broader patient‐specific objectives and desired outcomes for their medication, particularly within populations with multiple comorbidities and resultant polypharmacy. Therefore, there is a recent shift within the literature towards a goal‐orientated approach. This person‐centred design involves gaining a comprehensive understanding of individual patient needs within a medication review and aligning their needs with their specific goals for treatment. A goal‐orientated approach enables greater autonomy and facilitates shared decision making between the patient and the professional undertaking the review. This may lead to improvements in clinical, medication‐related, patient‐important and experience‐related outcomes.^47^, ^48^

Most of the existing literature and guidelines describe a disease‐orientated approach to medication prescribing for people living with CKD stage G4‐5D.^51^ Recent studies highlight the multitude of comorbidities in the CKD stage G4‐5D population.^52^ Various studies explain the significant burden arising from these multiple prescriptions.^4^, ^53^ These problems are not limited to individual medicines but are compounded by interactions between medicines and interactions with conditions that are becoming increasingly difficult to manage.

People living with CKD stage G4‐5D are particularly vulnerable to complex combinations of medicines.^12^, ^54^ These complexities include exposure to increased central nervous system effects, fall risk, bleeding risk, hypoglycaemia risk, anticholinergic burden and cardiovascular risk (QT prolonging) of sudden death.^55^ Patients with CKD stage G4‐5D have one of the highest polypharmacy rates, exceptionally high frailty rates,^2^, ^56^ susceptibility to side effects, have been excluded from most clinical trials^57^ and have increased adverse events,^53^ which raises the importance of medication review in this population.

The KDIGO 2024 Clinical Practice Guideline for the Evaluation and Management of Chronic Kidney Disease describes medication reviews as essential within the CKD G4‐5D population detailing the importance of a person‐centred approach.^58^ However, this review shows that how and when they should be offered, who should be prioritized, and the effect upon outcomes must still be understood.

### 4.3 Strengths and weaknesses

This review is the first to assess the extent of the literature describing interventions designed to address polypharmacy in people living with CKD stage G4‐5D. Our robust search strategy had no restrictions on language, population or publication date. The interventions were divided into themes including population characteristics, type and medicine classes. By revealing previously identified gaps in the literature, we strengthen the recommendations for future research.

This review focuses on the term medication review. This term was first conceptualized in the late 1990s with different levels defined in 2002 in the publication *Room for Review*.^59^ We have included the more recent enhanced classification of medication review, defined as disease‐orientated or goal‐orientated.^47^, ^48^

Medication review was used as a term to capture all studies focused on solutions to polypharmacy for advanced CKD stage G4‐5D.^44^ Our research omits studies describing polypharmacy, focusing instead on the studies that are investigating medication review as a solution to polypharmacy for advanced CKD stage G4‐5D.

We cannot comment on the quality of the identified literature as no quality assessment was conducted. We focused on the types of outcomes that researchers felt were important to measure in the context of the intervention rather than describing the totality of outcome assessments.

### 4.4 Gaps and directions for future research

This scoping review shows the lack of evidence in the current literature and highlights the need for more high‐quality studies to determine how to optimally deliver SMRs for people living with CKD stage G4‐5D. Future robust research should investigate how an SMR service can be co‐designed for patients living with CKD stages G4‐5D and polypharmacy and implemented in routine nephrology practice. Specifically, we aimed to identify key moments for optimal intervention and understand how to offer this service to reduce health inequalities in patients with CKD stage G4‐5D and polypharmacy.

Future studies should monitor the effects of SMR on short‐, medium‐ and long‐term clinical, patient‐important, medication‐related and participant‐related outcomes. Clinical outcomes of SMR should be based on 30‐day readmission and frailty scores, while patient‐important outcomes should include effects on QoL, and medication‐related outcomes could include monitoring the complexity of regimes against National Health Service polypharmacy indicators. All these outcomes should have healthcare‐related and medication‐related costs threaded to understand the financial impact.

A person‐centred SMR service designed for people living with CKD stage G4‐5D should be tested in future multi‐centre RCTs.

## 5 CONCLUSION

This scoping review highlights the multiple ways SMR interventions have been designed and studied in people living with CKD stages G4, G5, including those receiving dialysis, the majority by renal clinical‐pharmacists. There is a suggestion that such an intervention may improve outcomes for patients as is seen in those with other chronic illnesses. The KDIGO Chronic Kidney Disease guidelines describe the unique contribution of clinical pharmacists to medication‐related patient safety and outcomes. They also recommend a person‐centred approach to SMR for people with CKD and associated health problems.^58^ Renal clinical‐pharmacists are well placed to build on existing work, with future efforts focusing on increasing our understanding on how to optimize delivery of SMR interventions for the CKD G4‐5D population so patients can obtain maximum benefit from medication whilst harm is minimized.

## AUTHOR CONTRIBUTIONS

C.P. conceived the review, performed screening, data extraction and analysis, and drafted the manuscript. C.P. developed a search strategy supported by the Clinical Librarians at Portsmouth Hospitals University NHS Trust (R.H. and A.R.). R.C.A. screened all the search results and checked 10% of the extracted data. All authors contributed to the study and data analysis, and revised and approved the submitted manuscript.

## CONFLICT OF INTEREST STATEMENT

The authors report no conflicts of interest.

## Data Availability

Data supporting this study are openly available from the Open Science Framework (OSF.IO/HSW96; https://osf.io/hsw96).

## APPENDIX A

### SCOPING REVIEW SEARCH STRATEGY (20.10.2023)

**PCC—**Does ‘medicines optimization’ improve outcomes for people living with advanced chronic kidney disease?

### TOTAL REFERENCES IDENTIFIED

**529** (after 72 duplicates removed) ‐ Medline: 112, Embase: 352, CINAHL: 110, Cochrane: 27

**Inclusion criteria ‐** A description of inclusion principles based on SPIDER (Sample, Phenomenon of Interest, Design, Evaluation, Research type) criteria is provided in Table 1.

**Exclusion criteria ‐** Studies that included people on the transplant waiting list with a limited life expectancy due to other life‐limiting illnesses or acute kidney injury were excluded.

### ACCEPTED FOR ANALYSIS: 21

Major Themes Identified:

- Medication review or targeted deprescribing approaches
- Inconsistent measurements of clinical, patient‐important and medication‐ related outcomes
- Qualitative aspects of medication review or targeted deprescribing in chronic kidney disease

**Table:**

		Ovid MEDLINE (R) <1946 to October 18, 2023>	Ovid Embase (R) <1974 to October 18, 2023>	CINAHL (R) <1991 to October 18, 2023>
1	Renal Dialysis/	102 624	3269	15 221
2	Renal Insufficiency, Chronic/	36 562	1421	9019
3	Kidney Transplantation/	105 648	134 585	13 781
4	Kidney Failure, Chronic/	101 301	148 800	31 335
5	**1 OR 2 OR 3 OR 4**	**276 270**	276 425	51 938
6	Morbidity/	34 365	412 063	126 122
7	Comorbidity/	125 122	392 710	113 730
8	**6 OR 7**	**159 023**	787 014	230 567
9	Drug‐Related SE & ADRs/*	38 517	1751	22
10	Polypharmacy/	6742	24 037	7909
11	**9 OR 10**	**44 331**	25 718	7931
12	Medication Review/ref	132	4127	1616
13	Drug Utilization Review/	3880	1833	4555
14	Deprescriptions/	1042	1763	314
15	**12 OR 13 OR 14**	**5045**	6880	6351
**Combining Searches**				
16	**5 AND 8 AND 11**	**60**	**244**	**54**
17	**5 AND 15**	**53**	**124**	**58**
**Searches Results**				
18	**16 OR 17**	**112**	**352**	**110**

* Drug‐related side effects and adverse reactions.

**Table:**

	Cochrane: central register of controlled trials
“Renal dialysis” or “Renal Insufficiency, Chronic” or “Kidney Transplantation” or “Kidney Failure, Chronic” **AND** “Morbidity” or “comorbidity” **AND** “Drug‐Related Side Effects and Adverse Reactions” or “polypharmacy”	12
“Renal dialysis” or “Renal Insufficiency, Chronic” or “Kidney Transplantation” or “Kidney Failure, Chronic” **AND** “Medication Review” or “Drug Utilization Review”	15
**Search Results**	**27**

## APPENDIX B

### PROTOCOL CHANGES

Changes to the protocol resulted from the research team's assessment of identified eligible studies.

- Changing the inclusion criteria to allow studies which included CKD stage G1–3 if over 50% of the participants had CKD stage G4‐5D.
- Independent extraction of data by the two authors (CP and RA) was changed to a 10% check (by RA) for extraction by the main author (CP).

## APPENDIX C

### INCLUDED STUDIES

**Table:**

Covidence	Study no	Reference
480	1	Aghili M, Kasturirangan MN. Management of drug–drug interactions among critically ill patients with chronic kidney disease: impact of clinical pharmacist's interventions. *Indian Journal of Critical Care Medicine*. 2021;25(11):1226–1231. doi:10.5005/jp‐journals‐10 071‐23 919
306	2	Ahmed A, Blackburn DF, Evans C, Rosaasen N, Mansell H. The Saskatchewan Medication Assessment Program for patients with renal failure: a qualitative study to understand health care provider perspectives. *Canadian Journal of Kidney Health and Disease*. 2020;7:205435812095402. doi:10.1177/2054358120954028
86	3	Belaiche S, Romanet T, Allenet B, Calop J, Zaoui P. Identification of drug‐related problems in ambulatory chronic kidney disease patients: a 6‐month prospective study. *Journal of Nephrology*. 2012;25(5):782–788. doi:10.5301/jn.5000063
15	4	Bondurant‐David K, Dang S, Levy S, et al. Issues with deprescribing in haemodialysis: a qualitative study of patient and provider experiences. *International Journal of Pharmacy Practice*. 2020;28(6):635–642. doi:10.1111/ijpp.12674
517	5	Chia BY, Cheen MH, Gwee XY, et al. Outcomes of pharmacist‐provided medication review in collaborative care for adult Singaporeans receiving hemodialysis. *International Journal of Clinical Pharmacy*. 2017;39(5):1031–1038. doi:10.1007/s11096‐017‐0528‐1
544	6	Dashti‐Khavidaki S, Sharif Z, Khalili H, et al. The use of pharmaceutical care to improve health‐related quality of life in hemodialysis patients in Iran. *International Journal of Clinical Pharmacy*. 2013;35(2):260–267. doi:10.1007/s11096‐012‐9748‐6
9	7	George JS, Joseph R, Thomas ET, John GP, Siby A, Nair MM. Active deprescribing program in chronic kidney disease patients undergoing haemodialysis. *Nephrology*. 2021;26(11):890–897. doi:10.1111/nep.13936
7	8	Gerardi S, Sperlea D, Levy SO‐L, et al. Implementation of targeted deprescribing of potentially inappropriate medications in patients on hemodialysis. *American Journal of Health‐System Pharmacy*. 2022;79(Supplement 4):S128‐S135. doi:10.1093/ajhp/zxac190
111	9	Grabe DW, Low CL, Bailie GR, Eisele G. Evaluation of drug‐related problems in an outpatient hemodialysis unit and the impact of a clinical pharmacist. *Clinical Nephrology*. 1997;47(2):117–121. doi:https://pubmed.ncbi.nlm.nih.gov/9049460/
1	10	Hall RK, Rutledge J, Lucas A, et al. Stakeholder perspectives on factors related to deprescribing potentially inappropriate medications in older adults receiving dialysis. *Clinical Journal of the American Society of Nephrology*. 2023;18(10):1310–1320. doi:10.2215/cjn.0000000000000229
266	11	Kim AJ, Lee H, Shin E‐J, et al. Pharmacist‐led collaborative medication management for the elderly with chronic kidney disease and polypharmacy. *International Journal of Environmental Research and Public Health*. 2021;18(8):4370. doi:10.3390/ijerph18084370
583	12	Manley HJ, Aweh G, Weiner DE, et al. Multidisciplinary medication therapy management and hospital readmission in patients undergoing maintenance dialysis: a retrospective cohort study. *American Journal of Kidney Diseases*. 2020;76(1):13–21. doi:10.1053/j.ajkd.2019.12.002
165	13	Marquito AB, Pinheiro HS, Fernandes NM, Paula RB. Pharmacotherapy assessment in chronic kidney disease: validation of the pair instrument for use in Brazil. *Brazilian Journal of Nephrology*. 2020;42(4):400–412. doi:10.1590/2175‐8239‐jbn‐2019‐0205
598	14	Mateti U, Nagappa A, Attur R, Nagaraju S, Rangaswamy D. Impact of pharmaceutical care on clinical outcomes among hemodialysis patients: a multicenter randomized controlled study. *Saudi Journal of Kidney Diseases and Transplantation*. 2018;29(4):801–808. doi:10.4103/1319‐2442.239639
45	15	McIntyre C, McQuillan R, Bell C, Battistella M. Targeted deprescribing in an outpatient hemodialysis unit: a quality improvement study to decrease polypharmacy. *American Journal of Kidney Diseases*. 2017;70(5):611–618. doi:10.1053/j.ajkd.2017.02.374
601	16	Pai AB, Boyd A, Chavez A, Manley HJ. Health‐related quality of life is maintained in hemodialysis patients receiving pharmaceutical care: a 2‐year randomized, controlled study. *Hemodialysis International*. 2009;13(1):72–79. doi:10.1111/j.1542‐4758.2009.00328.x
25	17	Parker K, Bull‐Engelstad I, Benth JŠ, et al. Effectiveness of using STOPP/start criteria to identify potentially inappropriate medication in people aged ≥ 65 years with chronic kidney disease: a randomized clinical trial. *European Journal of Clinical Pharmacology*. 2019;75(11):1503–1511. doi:10.1007/s00228‐019‐02727‐9
338	18	Tesfaye WH, Wimmer BC, Peterson GM, et al. Effect of pharmacist‐led medication review on medication appropriateness in older adults with chronic kidney disease. *Journal of Pharmacy Practice and Research*. 2019;49(5):471–476. doi:10.1002/jppr.1539
371	19	Tuttle KR, Alicic RZ, Short RA, et al. Medication therapy management after hospitalization in CKD. *Clinical Journal of the American Society of Nephrology*. 2018;13(2):231–241. doi:10.2215/cjn.06790617
216	20	Moryousef J, Bortolussi‐Courval É, Podymow T, Lee TC, Trinh E, McDonald EG. Deprescribing opportunities for hospitalized patients with end‐stage kidney disease on hemodialysis: a secondary analysis of the MedSafer cluster randomized controlled trial. *Canadian Journal of Kidney Health and Disease*. 2022;9:20543581221098776–20 543 581 221 098 788. doi:10.1177/20543581221098778
96	21	Mirkov S. Implementation of a pharmacist medication review clinic for haemodialysis patients. *New Zealand Medical Journal*. 2009;122(1297):25–37.

## APPENDIX D

### Extraction template

**Table:**

Administrative			Context								Population			Concept			Results/findings				Gaps in evidence
Number	Author	Date of Publication	Country of Origin	Study Design	Single or multi‐centre	Prospective or retrospective	Total trial duration (months)	Description of setting (e.g. primary, secondary, older‐person, nephrology)	Profession of person conducting the study	Total number of participants	Age (yrs.) Mean (SD)	Renal function staging of participants (CKD 4, CKD5, CKD5D)	If CKD5D ‐ HD or PD	Medicine review or targeted deprescribing theme	Names of medicines described in study	Description of intervention (detail to include any validated frameworks)	Description of any patient‐related outcomes in reporting	Description of medication‐related outcomes in reporting	Description of qualitative outcomes in reporting	Follow‐up (months)	Research gaps identified

Abbreviations: CKD, chronic kidney disease; HD, haemodialysis; PD, peritoneal dialysis.

## APPENDIX E

**Table:**

Covidence	Study no.	Year of publication	Country of origin	Study design	Total no. of participants
598	14	2018	India	Prospective randomized controlled trial	200
25	17	2019	Norway	Prospective randomized controlled trial	180
371	19	2018	America	Prospective randomized controlled trial	141
601	16	2009	America	Prospective randomized controlled trial	107
216	20	2022	Canada	Secondary analysis of randomized controlled trial	140
544	6	2012	Iran	Prospective observational cohort study	92
583	12	2020	America	Retrospective observational cohort study	726
517	5	2017	Singapore	Retrospective observational cohort study	324
9	7	2021	India	Prospective observational case series	150
480	1	2021	India	Prospective observational case series	91
86	3	2012	France	Prospective observational case series	67
7	8	2022	Canada	Prospective observational case series	66
96	21	2009	New Zealand	Prospective observational case series	64
111	9	1997	America	Prospective observational case series	45
45	15	2017	Canada	Prospective observational case series	35
338	18	2019	Australia	Retrospective observational case series	204
165	13	2020	Brazil	Retrospective observational case series	100
266	11	2021	South Korea	Retrospective observational case series	95
1	10	2023	America	Qualitative study – rapid qualitative analysis	76
306	2	2020	Canada	Qualitative study – qualitative descriptive approach	28
15	4	2020	Canada	Qualitative study – grounded theory analysis	10

## APPENDIX F

**Table:**

Study no.	Year of publication	Theme	Clinical	Patient‐important	Medication‐related	Qualitative
9	1997	Medication review	Adverse drug reactions		Mean (SD) number of medicines before/after intervention Mean number of medicines stopped or started per patient Number of DRP identified and ranked. Acceptance rates by nephology	
16	2009	Medication review	Hospitalization rates	Renal QoL	Number of medicines	
21	2009	Medication review	Adverse drug reactions Extra monitoring Extra blood tests		Total number of interventions Mean number of interventions per patient Number of DRP identified, mean per patient	
3	2012	Medication review			Total number of interventions Number of DRP identified	Patient knowledge of nephroprotective medicines and renal risk situations. Self‐medication with OTC medicines (including NSAIDs) Request for help with medicines management.
6	2012	Medication review		Health related QoL SF‐36	Total number of interventions Acceptance rates by nephology	
5	2017	Medication review	Unplanned hospital admissions If admitted, duration of admission Mortality (not affected) Healthcare costs		Number of DRP identified No. of interventions	
15	2017	Targeted deprescribing		Patient satisfaction	Mean (SD) number of medicines before and after intervention Deprescribing success by class of medication	
14	2018	Medication review		Kidney Disease QoL‐36		
19	2018	Medication review	Composite outcome ‐ first acute care events (hospitalization, emergency department and urgent care centre visits) within the 90‐day period after index hospitalization. Categories of primary diagnoses for readmission.		Mean number of DRP identified Mean number of DRP resolved	
17	2019	Medication review		Medication adherence HRQoL	Potentially inappropriate medications (prescribed and omitted) according to STOPP/START version 2	
18	2019	Medication review			Medication appropriate index	
2	2020	Medication review				Community pharmacists' express pride about their work Renal physicians and pharmacists expressed concern over community pharmacists' recommendations
4	2020	Targeted deprescribing				Ambivalence towards dialysis and medication Empowerment through deprescribing The uncertain future of deprescribing clinics

12	2020	Medication review	Re‐admissions within 30 days after index hospitalization. Adverse drug events			
13	2020	Targeted deprescribing			Mean number of DRPs identified per patient.	
1	2021	Medication review	Extra monitoring Extra blood tests		changing dose, formulation, instructions for drug use, discontinuing medicine	
7	2021	Targeted deprescribing		Living with Medicines visual analogue score Medication adherence	Mean (SD) number of medicines per patient before/after intervention Mean pill‐count per day before/after intervention	
11	2021	Medication review			Mean (SD) of number of medications before/after intervention Number of potentially inappropriate medicines (PIMs) per patient before/after intervention. Potentially inappropriate medications (prescribed and omitted) according to STOPP/START version 2 Korean Anticholinergic Burden Scale (KABS)	
8	2022	Targeted deprescribing	Gout flares after stopping allopurinol BP and hyperkalaemia related to furosemide and alpha‐blocker deprescribing	Patient satisfaction	Mean (SD) number of medicines per patient before intervention Number (%) of target medicines per patient before intervention Number (%) of target medicines classified as inappropriate Number (%) of inappropriate target medicines successfully deprescribed, specific to patient and drug classification	
20	2022	Targeted deprescribing	Readmission rates Adverse drug reactions GI bleed rates		Percentage taking more than 10 regular meds. Percentage taking 1 or more potentially inappropriate medications categorized through expert consensus agreement	
10	2023	Targeted deprescribing		Symptom control prioritised by patients over potential harm from medicines		System‐level barriers to prescribing ‐ limited electronic interoperability Time‐constraints & competing priorities Undefined co‐management among clinicians ‐ unclear roles Limited knowledge about potentially inappropriate Medicines among clinicians and patients

DRP, drug‐related problem; GI, gastrointestinal; HRQoL, health‐related quality of life; NSAID, non‐steroidal anti‐inflammatory drug; OTC, over‐the‐counter; SD, standard deviation.
